# A Global Assessment of Coagulation Profile and a Novel Insight into Adamts-13 Implication in Neonatal Sepsis

**DOI:** 10.3390/biology12101281

**Published:** 2023-09-26

**Authors:** Paraskevi Papadogeorgou, Theodora Boutsikou, Maria Boutsikou, Eleni Pergantou, Aimilia Mantzou, Ioannis Papassotiriou, Zoi Iliodromiti, Rozeta Sokou, Elena Bouza, Marianna Politou, Nikoletta Iacovidou, Serena Valsami

**Affiliations:** 1 Neonatal Department, Medical School, National and Kapodistrian University of Athens, Aretaieio Hospital, 115 28 Athens, Greece; 2 Haemostasis Unit/Haemophilia Centre, “Aghia Sophia” Children’s Hospital, 115 27 Athens, Greece; 3 First Department of Paediatrics, Medical School, National and Kapodistrian University of Athens, “Aghia Sophia” Children’s Hospital, 115 27 Athens, Greece; 4 2nd Neonatal Intensive Care Unit, “Aghia Sophia” Children’s Hospital, 115 27 Athens, Greece; 5 Blood Transfusion Department, Aretaieio Hospital, Medical School, National and Kapodistrian University of Athens, 115 28 Athens, Greece

**Keywords:** neonates, sepsis, coagulation factors, anticlotting mechanism, ADAMTS-13, D-dimers

## Abstract

**Simple Summary:**

Coagulation and inflammation are two highly interrelated processes. The scope of the present study was to investigate the alterations of clotting and anticlotting mechanisms in neonates suffering from sepsis. Coagulation factors, natural inhibitors of coagulation and ADAMTS-13 (A disintegrin and metalloprotease with thrombospondin type-1 motives) were measured in term and preterm neonates in the acute phase of infection and after recovery. A control group of healthy neonates was also included. The results addressed by our study indicate a critical impairment of the hemostatic profile in neonates in the acute phase of infection, compared to recovery and the control group. Particular results are of great significance, as they are demonstrated for the first time and may be useful for the development of new therapeutic options in neonatal sepsis.

**Abstract:**

Neonatal sepsis is a life-threatening condition associated with significant morbidity and mortality. Sepsis-induced coagulopathy is a well-recognized entity, signifying the strong cross-talk between inflammation and coagulation. The aim of the present study was to compare the coagulation profile between the acute phase of sepsis and recovery in term and preterm neonates. Additional comparisons to healthy neonates were undertaken. Levels of clotting, anti-clotting factors and ADAMTS-13 (A disintegrin and metalloprotease with thrombospondin type-1 motives), the cleaving protein of von Willebrand factor (VWF), were measured in 16 term and preterm neonates in the acute phase of infection and following recovery, as well as in 18 healthy neonates. Clotting times were prolonged, while levels of particular clotting factors were lower in the acute phase of infection compared to controls and recovery. On the other hand, levels of fibrinogen, factor VIII (FVIII) and VWF were significantly higher in the acute phase in comparison to controls and recovery, while they remained persistently higher in the infection group compared to controls. In regard to the anticlotting mechanism, a clear suppression was observed in septic neonates. ADAMTS-13 levels were significantly lower in the acute phase of infection in comparison to controls and recovery (*p* = 0.015 and 0.004, respectively), while a trend toward superimposed normalization was demonstrated post infection, as higher ADAMTS-13 levels were measured in recovered neonates compared to controls (*p* = 0.002). The coagulation profile is considerably deranged in neonatal sepsis. ADAMTS-13 deficiency in septic neonates is a novel finding with promising future implications, as ADAMTS-13 substitution may serve as a useful therapeutic option in neonatal sepsis, prompting further investigation in future studies.

## 1. Introduction

Neonatal sepsis is a life-threatening condition, associated with significant morbidity and mortality because of neonates’ diminished innate and adaptive immune responses compared to adults. It is estimated that approximately 22/1000 live births are complicated by sepsis and the case mortality is 11–19% of those affected [1]. Worldwide, neonatal sepsis is associated with 15.6% of neonatal mortality [2]. The term “neonatal sepsis” refers to sepsis presenting in the first 28 days of life and is further classified into Early Onset Sepsis (onset of symptoms in the first 72 h after birth) and Late Onset Sepsis (presentation after 72 h of life). Despite the high burden of disease, there is no consensus definition of neonatal sepsis at present, and in clinical practice the diagnosis is based on microbiological cultures, clinical signs of infection and additional laboratory data [3,4].

Infection is a well-recognized trigger of the coagulation cascade. Inflammation and coagulation are strongly interrelated and the concept of “immunothrombosis” or “thromboinflammation” has arisen recently and was further reinforced during the COVID-19 pandemic [5]. Monocytes/macrophages respond to inflammation and express many types of receptors, such as Toll-like receptors (TLRs), which bind to the pathogen-associated molecular patterns (PAMPs), resulting in the activation of both the innate immunity and coagulation processes. Moreover, TLRs bind to host-derived stress molecules, particularly damage-associated molecular patterns (DAMPs), and monocytes are activated [6,7]. Proinflammatory cytokines released by monocytes, such as interleukin-6 (IL-6), induce the expression of tissue factor (TF) and phosphatidylserine (PS) on the cellular membrane and subsequently lead to full activation of the coagulation process [7,8]. Moreover, activated neutrophils release neutrophil extracellular traps (NETs). NETs, DAMPs and other cytotoxic molecules induce both inflammation and coagulation [6,7]. Platelets are also activated by inflammatory stimuli, augmenting platelet aggregation, assembly of clotting factors and adhesion of platelets to leukocytes [9]. Contrarily, activated platelets and coagulation factors interplay with the immune system in a variety of types, modulating the inflammatory response. Thrombin represents the major coagulation factor that regulates the inflammatory and coagulation processes by binding to protease-activated receptor-1 (PAR-1), expressed on monocytes, neutrophils, platelets and endothelial cells [10,11]. However, the activation of the coagulation process under infectious conditions is certainly not harmful but rather represents a host defense mechanism. The pathogens are entrapped inside the fibrin clot and their widespread dissemination is blocked. Furthermore, the activated coagulation system acts as an adjunct in the recognition of pathogens, recruitment of leukocytes, generation of antibacterial peptides and finally the killing of offending microorganisms [9]. Nevertheless, overactivation of the clotting process may lead to thrombosis, disseminated intravascular coagulation, bleeding and tissue damage.

The aim of the present study is to investigate the derangement of the hemostatic mechanism in term and preterm neonates suffering from early or late onset sepsis and compare it to healthy newborns.

## 2. Materials and Methods

This is a prospective cohort study of term and preterm neonates divided into two groups: those with early or late onset sepsis (infection group) and those serving as controls (control group). Newborns in the infection group were hospitalized at the 2nd Neonatal Intensive Care Unit, “Aghia Sophia” Children’s Hospital, and coagulation parameters were tested at two distinct time points: on the first day of infection onset and directly after the appropriate antibiotic course was terminated. The control group consisted of healthy neonates born at Aretaieio Hospital, National and Kapodistrian University of Athens. Exclusion criteria for the control group included: maternal thrombophilia or any other maternal coagulation disorder, maternal intake of anticoagulation treatment, aspirin and antiepileptic medication, diabetes (gestational, type 1 or 2), preeclampsia, maternal lupus erythematosus, perinatal stress (defined as Apgar score 5′ < 7) and fetal-growth-restricted neonates, as well as neonates with cardiorespiratory disorders, infection and congenital anomalies. The study period extended from May 2017 to July 2020. The Ethics Review Committees of both hospitals approved the study and parents gave informed consent prior to recruitment. The infection group consisted of 16 neonates, while the control group consisted of 18 neonates. Hemostatic profile comparisons were undertaken between the acute phase of infection and post infection, between the acute phase of infection and controls and between the recovery phase and controls.

Demographic data and perinatal parameters were collected from neonatal medical records. Infection definition was based on a combination of risk factors, clinical symptoms and signs at disease onset and during the following 36–48 h, in association with elevated infection biomarkers, mainly C-reactive protein and administration of antibiotics for at least ten days [12,13,14]. A positive blood culture was not deemed a prerequisite for the definition of infection because the infection group was derived from a tertiary NICU and most babies were referred from distinct hospitals and already on antibiotic treatment at the time of admission. Furthermore, maternal intrapartum antibiotic treatment may mask the blood culture results. Neonatal SOFA (nSOFA) scores were applied in the infection group to classify infection according to its severity [15,16]. Furthermore, the type of infection was classified as either Early-Onset Sepsis (<72 h of life) or Late-Onset Sepsis (≥72 h).

Blood samples in both groups were collected during assessment for clearly indicated medical conditions and newborns were not subjected to extra painful stimuli. Blood was collected in tubes containing citrate in the ratio 9:1. The coagulation parameters tested are as follows: Prothrombin Time (PT), Activated Partial Thromboplastin Time (APTT), Fibrinogen, Factors II, VII, VIII, IX, X, von Willebrand (VWF), Ristocetin cofactor activity (Rcof), Antithrombin (AT), Proteins C and S (PC and PS, respectively), D-Dimers, Platelets (PLT) and ADAMTS-13 protein (A disintegrin and metalloprotease with thrombospondin type-1 motives), the cleaving protein of VWF. The coagulation tests were performed in the Haemostasis Unit/Haemophilia Centre, “Aghia Sophia” Children’s Hospital. Platelet counts were derived from compete blood counts performed at the Hematology Laboratory, “Aghia Sophia” Children’s Hospital.

Statistical analysis was performed by SPSS (version 23; SPSS Inc., Chicago, IL, USA). One-way ANOVA was applied in order to detect differences in PT, APTT, Fibrinogen, FII, FVII, FVIII, FIX, FX, VWFAg, Rcof, AΤ, PC and PS, D-Dimers, PLT and ADAMTS-13 among the study groups. Student’s T test was used to examine differences in continuous variables among groups, while the paired T test was applied to examine differences in coagulation parameters between the acute phase of infection and post infection. Sensitivity and specificity were calculated for different values of ADAMTS-13. ROC analysis was performed to evaluate the performance of ADAMTS-13 in classifying neonates with acute infection and healthy controls. A receiver operator characteristic (ROC) curve was used to identify the best cutoff value of ADAMTS-13 that predicts acute infection. The X2 chi square test was applied to detect differences between categorical variables. Pearson’s correlation coefficient was used to examine possible correlations between continuous variables. *p* < 0.05 was considered statistically significant.

## 3. Results

Demographic data and perinatal parameters are presented in Table 1. All coagulation parameters were measured during the acute phase of infection and post infection. Sixteen neonates were enrolled in the infection group with mean gestational age 32.7 ± 2.9 weeks and mean birthweight 1644 ± 672 gr. Twelve neonates (75%) were premature, while four of them (25%) were born at term. Nine neonates (56.3%) were male and seven (43.8%) were female. Mean CRP values were 50.6 ± 77.7 mg/L. Positive blood culture was present in 6 cases (37.5%). Late Onset Sepsis was present in 9 cases (56.3%) and Early Onset Sepsis in the other 7 cases (43.8%). In the control group, 18 neonates were included, with mean gestational age 38.2 ± 1.5 weeks and mean birthweight 3090 ± 461 gr. Three (16.7%) neonates were premature, while 15 (83.3%) were full-term. Eight (44.4%) neonates in the control group were male and 10 (55.6%) were female. None of the infected neonates succumbed to their disease.

The values are presented as the mean and standard deviation or number of patients and percentages in brackets. *p* < 0.05 was considered statistically significant. Abbreviations: IVF, in-vitro fertilization; SGA, small for gestational age, Birthweight < 10th percentile; AGA, appropriate for gestational age, Birthweight ≥ 10th percentile.

(a) Comparison of coagulation parameters between acute phase of infection and healthy controls

**Figure 1:**
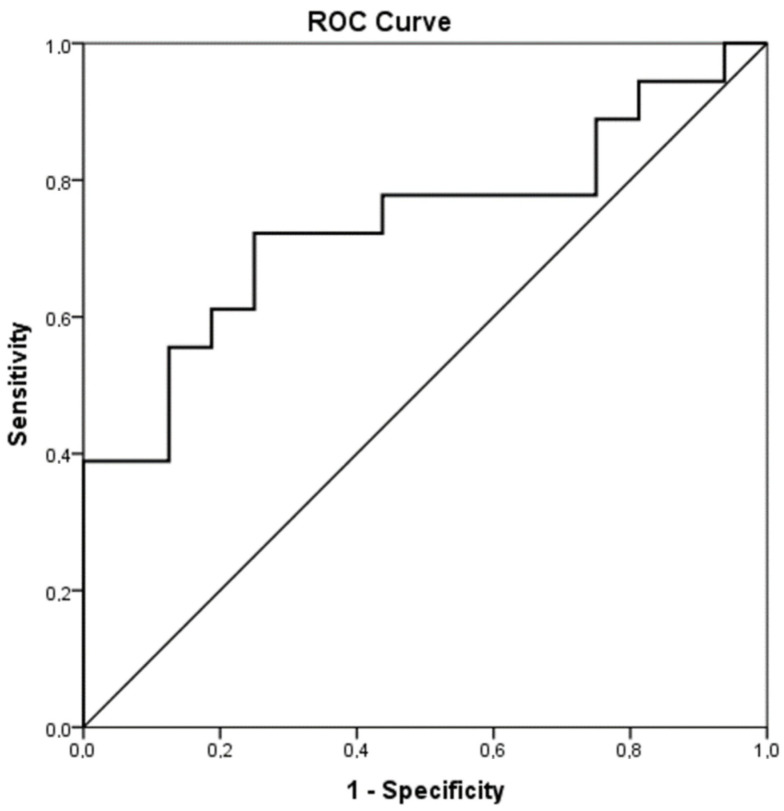
ROC analysis of ADAMTS-13 as a predictor of infection.

The hemostatic mechanism was considerably deranged in the acute phase of infection compared to the healthy controls. PT and APTT were prolonged in the infection group (acute phase) compared to the control group (15.2 ± 4.1 s vs. 11.9 ± 1.8 s, *p* = 0.014 and 33.1 ± 4.5 s vs. 29.5 ± 2.7 s, *p* = 0.040, respectively). What is more, levels of FII, FVII and PC were significantly decreased in the acute phase of infection compared to the controls (Table 2). FIX and AT were similarly more decreased in the infection group (acute phase) than in the controls, although the difference was not statistically significant. PS levels were almost equal between the infected neonates (acute phase) and the controls (Table 2). Contrarily, Fibrinogen, FVIII, VWF and Rcof were elevated in the infection group (acute phase) compared to controls and the difference was statistically significant. Interestingly, D-dimers were increased in the control group but not in the patient group, even though there was no statistically important discrepancy. ADAMTS-13 was decreased in the acute phase of infection in contrast to the controls (488.5 ± 75.4 ng/mL vs. 577.2 ± 113.6 ng/mL, *p* = 0.015). Figure 1 shows the ROC analysis of ADAMTS-13 as a predictor of infection (AUC = 0.736, CI95% 0.564–0.908, *p* = 0.019). The chosen cutoff value was 545.5 ng/mL, with sensitivity 72% and specificity 75%.

A significant negative correlation was observed between VWF and ADAMTS-13 in the acute phase of infection (r = −0.0575, *p* = 0.020).

(b) Comparison of coagulation parameters between acute phase of infection and recovery

Clotting times were prolonged in the acute phase of the disease in comparison to recovery, although only PT reached a statistically significant difference, *p* = 0.014. APTT values were also extended in the acute phase compared to recovery, *p* = 0.750 (Table 2). FVII, and the natural inhibitors of coagulation were substantially diminished in the acute phase of infection compared to recovery (Table 2). FVII levels were 36.1 ± 22.8% in the acute phase vs. 64.8 ± 15.2% post infection, *p* = 0.001. Additionally, levels of AT and PS were lower in the acute phase in comparison to recovery and the difference was statistically significant (*p* = 0.011 and *p* = 0.01, respectively). On the other hand, Fibrinogen, FIX, FVIII, VWF and Rcof at the first time point were elevated in comparison to recovery and all the values reached statistical significance (Table 2). D-dimers were increased while PLT count and ADAMTS-13 were found to be at lower levels in the acute phase compared to post infection and differences were statistically significant (Table 2).

(c) Comparison of coagulation parameters between recovery and healthy controls

In regard to hemostatic profile post infection in comparison to the control group, APTT remained slightly prolonged, *p* = 0.001. A trend towards normalization was demonstrated, although not to a full extent (Table 2). Exceptionally, FIX was decreased post infection compared to controls (*p* = 0.003), while PS was elevated (*p* = 0.034). VWF and Rcof remained elevated post infection compared to controls and the difference was statistically significant (Table 2). ADAMTS-13 increased post infection with levels exceeding controls (618.8 ± 130.0 ng/mL vs. 577.2 ± 113.6 ng/mL, *p* = 0.002).

In order to investigate other parameters that could impair the hemostatic profile, we tested possible variations between males and females and found that PT mean levels were considerably higher in females than in males (17.0 ± 4.1 s vs. 12.3± 1.2 s, *p* = 0.006). Accordingly, FVII mean levels were significantly lower in females than in males (25.4 ± 17.0% vs. 55.7 ± 12.7%, *p* = 0.001). The comparison between preterm and full-term newborns revealed that FIX and PC levels were significantly lower in preterm neonates (45.8 ± 16.1% vs. 68.8 ± 21.1%, *p* = 0.038 and 29.2 ± 8.4% vs. 42.3 ± 10.8%, *p* = 0.024, respectively). Possible correlations between coagulation parameters and birthweight were explored, but no significant differences were observed between SGA and AGA neonates. The severity of infection as assessed by nSOFA score was positively correlated with VWF levels in the acute phase of infection (r = 0.598, *p* = 0.014) and negatively correlated with ADAMTS-13 levels at the same time point (r = −0.531, *p* = 0.034). However, no significant difference was observed in respect to the type of offending pathogen, in cases in which this was isolated. No significant correlations were observed between ADAMTS-13 and VWF levels with respect to blood type. What is more, ADAMTS-13 levels were positively correlated with PLT count in the acute phase of infection (r = 0.507, *p* = 0.045) and negatively correlated with CRP (r = −0.787, *p* < 0.001). In regard to type of delivery and perinatal stress in the infection group, AT mean levels were significantly higher in neonates delivered by vaginal delivery (66.0 ± 11.0% vs. 49.2 ± 11.2%, *p* = 0.018), while FII mean levels were significantly elevated in cases of peripartum stress (45.3 ± 5.4% vs. 53.6 ± 7.1%, *p* = 0.044).

Finally, the mean value of ADAMTS-13 in the control group was compared to adult values published in the literature [17] and was found to be significantly lower (*p* < 0.001).

In regard to clinical implications of the aforementioned coagulation derangement, no clinically apparent thrombotic episodes were observed. Bleeding complications were observed in 2 (12.5%) neonates, the first of minor severity, while the second preterm neonate suffered from disseminated intravascular coagulation (DIC) in the setting of Gram negative septicemia. A progression of a low grade intraventricular hemorrhage into a higher grade was also observed in the last case. Mortality rates were null.

## 4. Discussion

Our study indicates that the coagulation profile is considerably deranged in term and preterm neonates in the acute phase of infection in comparison to healthy controls and exerts potential normalization after recovery, although not to a full extent. In line with our results, several studies have demonstrated increased levels of fibrinogen, prolongation of PT and APTT and lower PLT counts in neonates and children with sepsis, in comparison to age-matched controls [18,19,20,21]. However, to the best of our knowledge this is the first study assessing ADAMST-13 levels in neonatal sepsis and exploring its crucial role in sepsis-induced coagulopathy in newborns.

Prolongation of clotting times is probably caused by the lower levels of coagulation factors observed in sepsis, which was also demonstrated in our study regarding FII and FVII. Interestingly, FIX levels were elevated, rather than decreased, in the acute phase versus post infection. However, FIX levels were diminished in neonates post infection in comparison to controls, probably indicating later impairment of FIX during the disease process, which remains low for a longer time. This pattern of impairment of coagulation factors (early for FII and FVII and delayed for FIX) probably reflects a defect in the vitamin K action mechanism. It is known that vitamin K deficiency is first accompanied by a reduction in levels of FVII, then FII and FX and finally FIX [22]. Vitamin K deficiency is further supported by lower levels of PC and PS. The pathogenesis of vitamin K deficiency in septic neonates is multifactorial and may still be present despite universal vitamin K prophylaxis at birth. First of all, vitamin K does not cross the placenta, resulting in relative deficiency, which is further exacerbated by inadequate bacterial gut colonization at birth. Breastfeeding is also a poor supplier of vitamin K, while prolonged antibiotic exposure, hyperalimentation and malabsorption are additional factors leading to vitamin K deficiency. Furthermore, vitamin K metabolism may be altered in sepsis [22,23,24]. The patient group of our study was exposed to all the aforementioned conditions. Additionally, lower FIX levels post infection in comparison to controls may be attributed to the higher percentage of premature babies included in the infection group. Nevertheless, lower levels of the particular coagulation factors in the acute phase of infection may also be ascribed to consumption coagulopathy during infection, an assumption further reinforced by the identification of elevated D-dimers and decreased PLT count at the first time point in comparison to recovery.

On the other hand, elevated fibrinogen levels in the acute phase of infection compared to recovery and controls are probably attributed to its action as an acute-phase reactant. Fibrinogen plays a key-role in host defense by acting as a chemotactical factor for monocytes and neutrophils, while it also participates in fibrin clot formation, where bacteria are trapped and immobilized. However, fibrinogen may be consumed during the progression of infection and has been linked to complicated sepsis, concurrent DIC and poor prognosis in neonates and children with sepsis [19,25,26].

**Figure 2:**
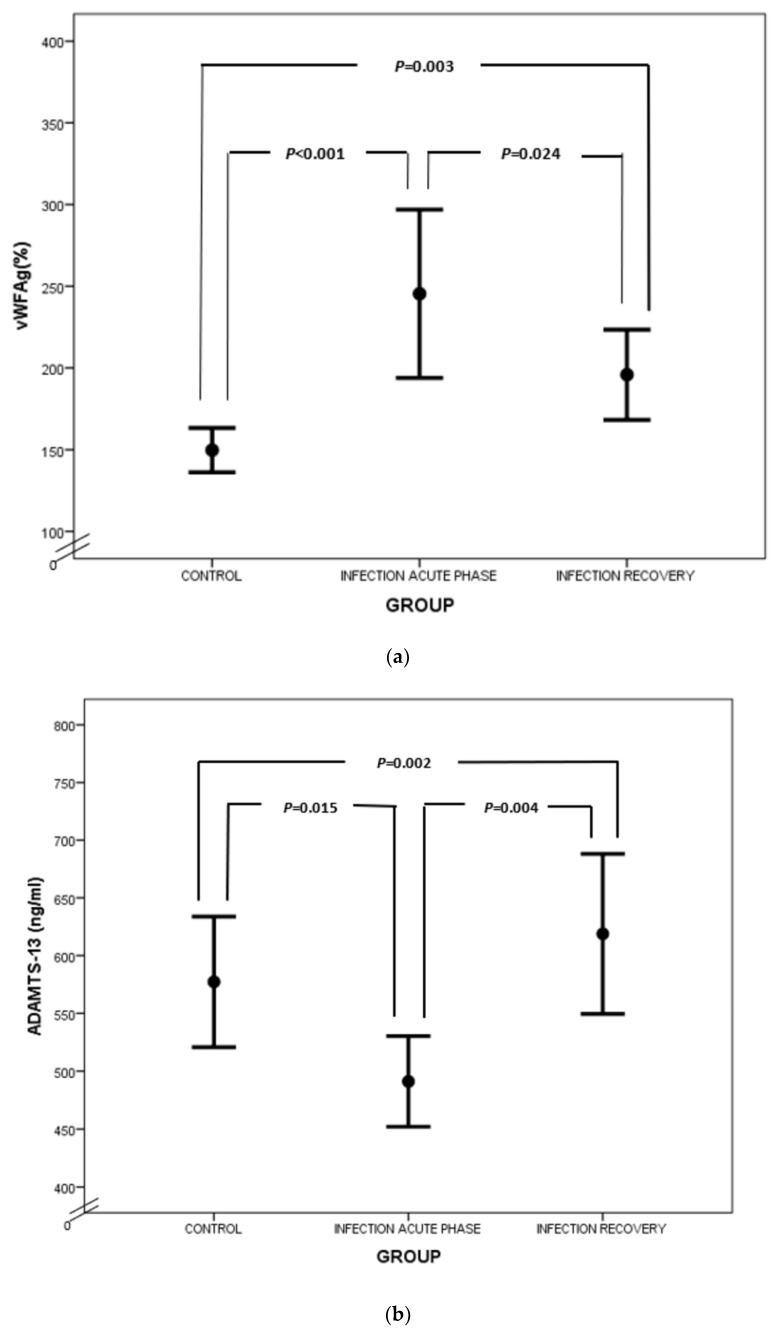
Comparison of VWF (**a**) and ADAMTS-13 (**b**) between controls, acute phase of infection and recovery.

**Figure 3:**
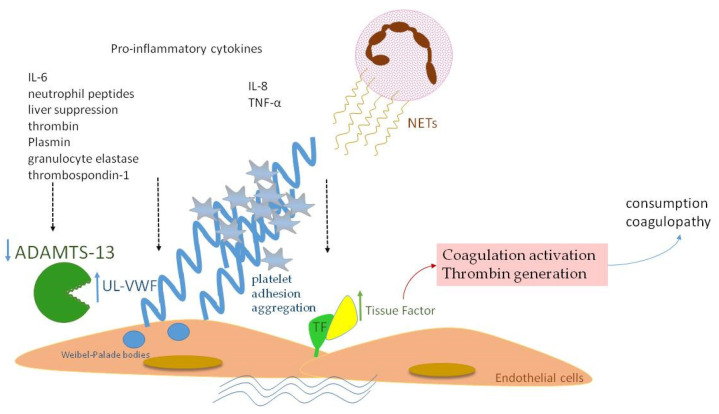
A schematic diagram of ADAMTS-13 implication in neonatal sepsis. Proinflammatory cytokines and NETs induce TF expression with subsequent full activation of the coagulation process. VWF is released in large amounts in the form of UL–VWF, whose size is normally regulated by ADAMTS-13. Lower levels of ADAMTS-13 in neonatal sepsis are attributed to: (a) inhibition by IL-6 and neutrophil peptides, (b) liver dysfunction, (c) degradation by thrombin, plasmin and granulocyte elastase, (d) competitive inhibition by thrombospondin-1.

Similar to fibrinogen, FVIII and VWF levels are elevated in sepsis, as they also serve as acute-phase reactants (Figure 2a). Enhanced production of FVIII is mediated by IL-6 [27]. VWF is produced mainly by endothelium and secondly by megakaryocytes. Endothelial activation during infection leads to release of VWF in large amounts, promoting aggregation of platelets, granulocytes and monocytes, as well as adhesion of bacteria like *Staphylococcus aureus* [28,29,30]. VWF in sepsis represents another component of “immunothrombosis”, as VWF interacts with NETs by electrostatic forces, further inducing the vicious cycle of inflammation and thrombosis [31]. VWF is released into circulation in the form of ultra-large multimers (UL–VWF), which are highly reactive and hyperadhesive, augmenting platelet adhesion and aggregation and finally clotting formation. Under flow conditions, the prothrombotic tendency of UL–VWF is downregulated by ADAMTS-13, the cleaving protein of UL–VWF multimers, which is produced by hepatic stellate cells and vascular endothelial cells [32]. ADAMTS-13 represents a negative acute-phase protein whose function is attenuated in cases of systemic inflammation due to several pathophysiologic mechanisms [33]. First of all, inflammatory mediators, such as Ιnterleukin-8 (IL-8) and Tumor Necrosis Factor-α (TNF-α), stimulate endothelial release of UL–VWF, while IL-6 and neutrophil peptides inhibit ADAMTS-13 action [34,35]. Additionally, the synthetic ability of the liver is suppressed during sepsis, while various proteases, such as thrombin, plasmin and granulocyte elastase, have been shown to degrade ADAMTS-13 [36]. The massive production of UL–VWF during sepsis may also lead to consumption of ADAMTS-13 [37]. What is more, oxidation associated with inflammation results in oxidative modification of VWF and ADAMTS-13. Oxidized VWF cannot be cleaved by ADAMTS-13, while oxidization of ADAMTS-13 leads to inhibition of its proteolytic activity [38]. Finally, high levels of thrombospondin-1 upon inflammation may theoretically result in reduced ADAMTS-13 activity, due to competitive inhibition of ADAMTS-13 binding to VWF [39]. Indeed, ADAMTS-13 levels were diminished in our patient group during the acute phase of infection in comparison to recovery (Figure 2b), as well as in comparison to controls. A schematic approach to ADAMTS-13 implication in neonatal sepsis is presented in Figure 3.

Decreased ADAMTS-13 levels have been previously described in several studies regarding adult and pediatric patients with sepsis [37,40,41,42,43,44,45,46,47,48,49,50,51]. ADAMTS-13 levels in sepsis have been measured at around 20–43% of normal levels and an imbalance in the VWF/ADAMTS-13 system is observed under inflammatory conditions [33,42]. Certain studies, similarly to ours, have demonstrated the persistence of high VWF levels during the course of sepsis, particularly in patients with the worst outcomes [42,43].

Interestingly, a trend towards superimposed normalization of ADAMTS-13 was demonstrated post infection. This finding may reflect a compensating mechanism to increased VWF concentrations later in the disease progress or a restoration of hepatic synthetic ability after recovery. However, this assumption needs further confirmatory data. The same trend was demonstrated by several studies conducted in pediatric and adult patients with severe sepsis [37,42,49]. In contrast to us, Singh et al. found persistently decreased ADAMTS-13 antigen and activity in adult ICU septic patients at discharge, a fact that may indicate sustained prothrombotic risk post infection in the adult population [46]. Claus et al. demonstrated significantly low ADAMTS-13 activity in adult septic patients, which increased in survivors rather than in non-survivors. In the same study, a subgroup of healthy individuals was subjected to strenuous exercise in order to induce uncomplicated systemic inflammatory response syndrome (SIRS). Surprisingly, they showed a positive correlation between ADAMTS-13 and VWF, suggesting a possible compensatory mechanism, active only in healthy individuals with moderate SIRS [43] The same compensatory mechanism may also justify our results. Furthermore, it is worth mentioning that increased ADAMTS-13 levels in our study population post infection may be attributed to developmental aspects of coagulation and further verify the principle that the hemostatic system is balanced and protected against thrombotic complications early in life [52]. Nevertheless, more data are required to verify this assumption. Developmental hemostasis is further reinforced by the lower ADAMTS-13 levels in the healthy control group in comparison to the adult levels published in the literature, in accordance with previous studies demonstrating neonatal ADAMTS-13 levels as low as 25–30% of adult levels [17,53]. It is worth noticing that, to the best of our knowledge, this is the first study assessing ADAMTS-13 levels in neonatal sepsis and elucidating the delicate implication of ADAMTS-13 in neonatal-sepsis-induced coagulopathy.

In addition, our study showed a positive correlation between ADAMTS-13 levels and platelet count that is consistent with the findings of other studies [37,40,43,44,45,54]. ADAMTS-13 deficiency is associated with thrombus formation and concurrent platelet consumption, while bone marrow becomes both exhausted and suppressed under septic conditions [44,55]. As expected, the PLT count was lower in the acute phase of infection in comparison to recovery. Platelet immunology has recently been introduced as an emerging topic and growing evidence indicates that platelets play a key-role in the host defense response. First of all, platelets promote leukocyte recruitment and activation while initiating clot formation, where pathogens are contained and trapped. Moreover, they interact directly with pathogens and even exert bactericidal activity against *Staphylococcus aureus* [56].

**Figure 4:**
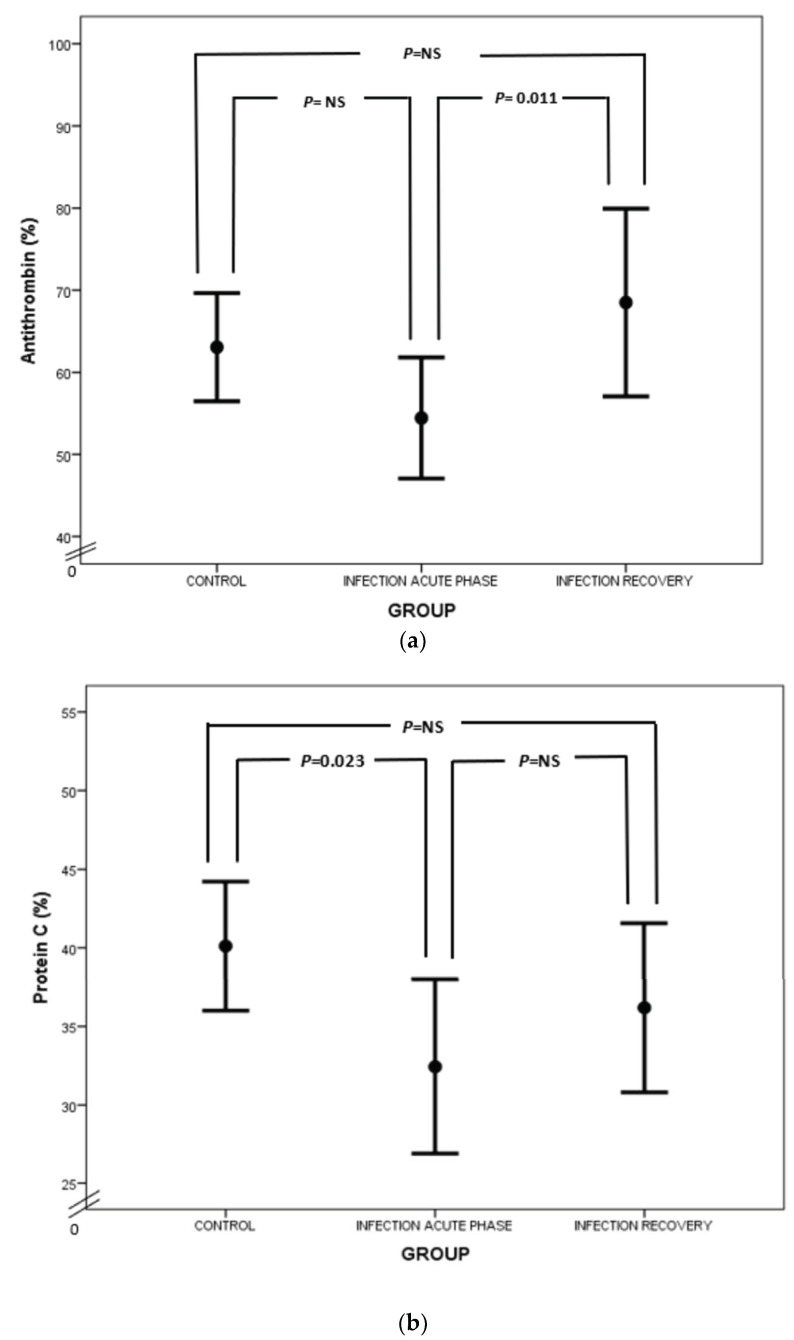

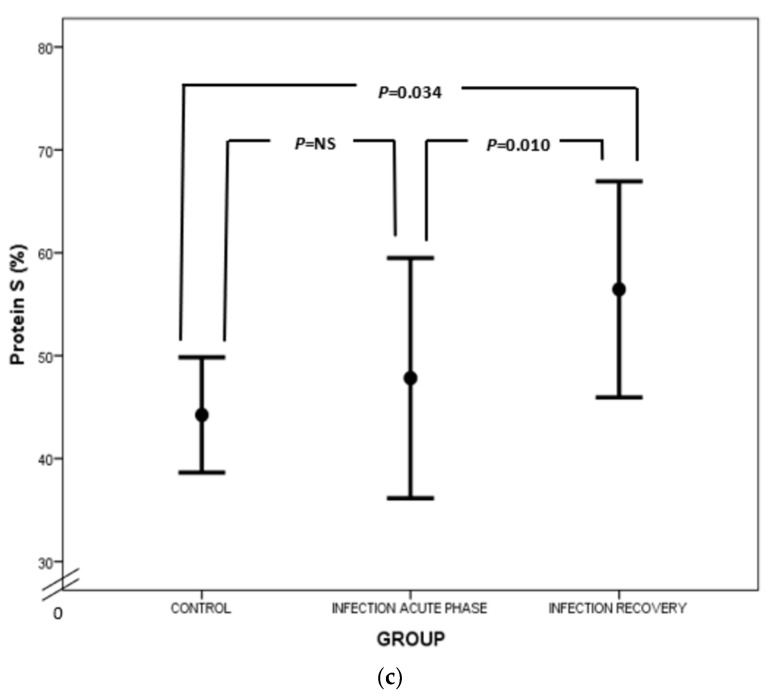
Comparison of AT (**a**), PC (**b**) and PS (**c**) values between acute phase of infection and recovery.

Considering natural anticoagulants (AT, PC and PS) in neonatal sepsis, our study demonstrated a suppression of this anticlotting mechanism. In particular, AT levels were lower in neonates with sepsis than in healthy neonates (Figure 4a), which is in accordance with previous reports [25,57]. AT exerts its anticoagulant activity by neutralizing thrombin and activated FX (FXa) and to a lesser extent activated FIX and FVII (FIXa and FVIIa, respectively) [58]. Lower levels of AT in sepsis are attributed to AT consumption in the activated coagulation process, impaired liver synthesis and AT inactivation by neutrophil-induced elastase. Syndecan shedding also contributes to AT decrease in sepsis [59,60,61]. Besides the net anticoagulant activity of AT, anti-inflammatory properties are also prominent. Endothelial prostacyclin, induced by AT, inhibits platelet aggregation and activation, neutrophil and blood vessel wall interaction, and cytokine production. Furthermore, direct interplay between AT, leukocytes and lymphocytes takes place, while AT inhibits thrombin’s implication in the inflammatory process [62].

Regarding PC, our study has highlighted PC deficiency in septic newborns, in accordance with the findings of other studies (Figure 4b). Protein C deficiency has been linked to shock, organ involvement and death in neonates as well as the adult and pediatric populations [20,63,64,65,66,67,68]. PC is another potent anticoagulant protein. It exerts its action by inhibiting activated FV and FVIII (FVa and FVIIIa, respectively) together with its cofactor, PS, while PC also neutralizes plasminogen activator inhibitor (PAI) [58,69]. PC’s activation is promoted by endothelial thrombomodulin (TM) and endothelial protein C receptor (EPCR). PC has also anti-apoptotic and anti-inflammatory properties by preventing endothelial apoptosis, enhancing endothelial barrier function and suppressing certain inflammatory genes [70]. Quantitative and qualitative abnormalities of PC and PS are observed under septic conditions due to liver dysfunction, altered vitamin K metabolism and consumption coagulopathy. Furthermore, PC is proteolyzed by granulocyte elastase and is also inadequately activated in sepsis. Additionally, increased PAI-1 levels inhibit PC function, while free and active PS is reduced due to elevated levels of C4b-binding protein [23]. In our study, total protein S levels were almost the same, rather than decreased, in infected neonates in comparison to controls (Figure 4c). In accordance with ourselves, Román et al. have found similar total PS levels in infected and healthy newborns, probably due to endothelial damage in sepsis leading to TM downregulation, suppressed PC activation and finally reduced PS consumption. However, free PS values were lower in infected neonates compared to controls (Figure 4c) [67]. Similar results have also been demonstrated in adult septic patients [71]. Noteworthily, the suppression of natural inhibitors in neonatal sepsis in our study was not associated with disease severity.

An intriguing result was the identification of lower D-dimer levels in the infection group compared to the control group. It is well known that D-dimer is produced by fibrin breakdown. Fibrinogen is converted to fibrin, which is then stabilized by activated FXIII and subsequently degraded by plasmin. D-dimer production necessitates the activation of both coagulation and fibrinolysis [72]. The control group demonstrated markedly high D-dimer levels, maybe as a result of coagulation activation during delivery and in line with several studies on developmental hemostasis showing equalization with adult values only at puberty [73,74,75,76,77]. In contrast to our results, other studies have shown significantly higher D-dimer levels in septic newborns and children compared to age-matched controls, although only full-term neonates were included [18,78,79,80,81,82]. On the other hand, Brahamna et al. encountered mainly preterm neonates and found low D-dimer levels in neonatal sepsis, in agreement with us [83]. The discrepancy between study results may reflect the pre-existing controversy about fibrinolysis with regard to gestational age. Some investigators have shown increased fibrinolytic activity with decreased gestational age, while others have found suppressed fibrinolysis in preterm rather than full-term neonates [84,85,86,87]. Nonetheless, fibrinolysis is downregulated under septic conditions, mainly due to the enhanced activity of Plasminogen Activator Inhibitor 1 (PAI-1) [88]. D-dimer levels in sepsis may be false negatives and may not totally reflect the magnitude of fibrin formation, raising concerns that the classical criteria used for diagnosis of DIC may be misleading [89].

Interestingly, recent studies doubt the traditional assumption that high D-dimer levels are associated with increased mortality in sepsis. Semerano et al. revealed that adult septic patients without D-dimer increase had a greater risk of mortality, probably due to of impaired fibrinolysis in sepsis [89,90]. Similarly, Han et al. demonstrated a U-shaped relationship between D-dimer levels and in-hospital mortality in adult septic patients [91]. In regard to children, Wang et al. demonstrated no association between D-dimer levels and in-hospital mortality in children with bacteremia, probably because of the different specificities of D-dimer with particular conditions and pathogens [92]. The role of D-Dimer in septic neonates needs further evaluation in large population studies.

Finally, the strong interplay between inflammation and the coagulation process was further verified by our study, as demonstrated by the clear association of nSOFA scores with VWF and ADAMTS-13 levels. Additional studies have shown that VWF and ADAMTS-13 levels are clearly associated with disease severity and/or poor prognosis in sepsis [37,40,41,42,43,45,47,48,49,93]. The underlying mechanism is probably inadequate cleavage of UL–VWF, leading to enhanced platelet–vessel wall interactions and subsequently to thrombotic microangiopathy, associated with thrombocytopenia, nonimmune hemolysis and end-organ damage [94]. Indeed, microthromboembolism is a common autopsy finding in cases of fatal septic shock [95]. In our study, no correlation with mortality could be undertaken due to absence of fatality.

Regarding limitations, our study’s sample size can be considered small, but the fact that it is well-defined and homogenous can lead to more valuable results. Developmental aspects of hemostasis due to inconsistency of gestational age between the patient and control groups cannot be ignored. During the study period, great concern was paid to avoid this fault, but preterm neonates commonly have morbidities that block them from serving as controls.

## 5. Conclusions

The present study represents a spherical assessment of the coagulation profile, evaluating the hemostatic process at two distinct time points and elucidating the evolution of coagulation abnormalities in neonatal sepsis. Conventional coagulation assays, as well as clotting and anticlotting factors, are greatly impaired in neonates under septic conditions, a fact with a major impact on clinical course and prognosis. It is well known that novel strategies are highly required in the battle against sepsis in order to facilitate improved survival and long-term outcome for the vulnerable neonatal population. To the best of our knowledge, this is the first study assessing ADAMTS-13 levels in neonates with sepsis. The confirmation of low levels of ADAMTS-13 in neonates with sepsis in further studies could be of great importance, since ADAMTS-13 substitution has been proposed as an adjuvant tool, currently under investigation, in sepsis treatment.

## Figures and Tables

**Table 1:** Demographic data.

Variable	Infection (Acute Phase)	Infection (Recovery)	Control	*p*
N	16	16	18	
Gestational age (weeks)	32.7 ± 2.9		38.2 ± 1.5	0.001
Chronological age (days)	11 ± 15	28.8 ± 17.3	4.6 ± 2.2	<0.001
Corrected age (weeks)	33.7 ± 4.3	36.0 ± 4.5		0.208
Birthweight (g)	1644 ± 672		3090 ± 461	0.001
Prematurity				0.001
Yes	12 (75%)		3 (16.7%)	
No	4 (25%)		15 (83.3%)	
Gender				0.366
Male	9 (56.3%)		8 (44.4%)	
Female	7 (43.8%)		10 (55.6%)	
Type of conception				0.742
Normal	15 (93.8%)		16 (94.1%)	
IVF	1 (6.3%)		1 (5.9%)	
Prolonged rupture of membranes (≥18 h)				0.146
Yes	4 (26.7%)		1 (6.3%)	
No	11 (73.3%)		15 (93.8%)	
Type of delivery				0.081
Vaginal delivery	5 (31.1%)		11 (61.1%)	
Cesarean section	11 (68.8%)		7 (38.9%)	
Perinatal stress	6 (37.5%)			
Resuscitation				<0.001
None	3 (18.8%)		18 (100%)	
Neopuff	9 (56.3%)			
Intubation	4 (25%)			
SGA				0.455
Yes	2 (12.5%)		1 (5.6%)	
No	14 (87.5%)		17 (94.4%)	

**Table 2:** Comparison between control and infection (acute phase and recovery) groups.

Variable	Control Mean ± SD	Infection (Acute Phase) Mean ± SD	Infection (Recovery) Mean ± SD	p^1^	p^2^	p^3^
Chronological Neonatal Age (days)	4.6 ± 2.2	11 ± 15	28.8 ± 17.3	-	-	-
Corrected neonatal age (weeks)		33.7 ± 4.3	36.0 ± 4.5	-	-	-
PT (s)	11.9 ± 1.8	15.2 ± 4.1	11.9 ± 0.8	**0.014**	0.979	**0.014**
APPT (s)	29.5 ± 2.7	33.1 ± 4.5	32.5 ± 2.2	**0.040**	**0.001**	0.750
Fibrinogen (mg%)	268.8± 55.8	379.6± 94.0	269.9 ± 63.1	**0.002**	0.957	**0.005**
FII (%)	59.8 ± 17.1	56.4 ± 15.6	54.1 ± 11.1	**0.044**	0.100	0.352
FVII (%)	60.1 ± 16.0	36.1 ± 22.8	64.8 ± 15.2	**0.009**	0.387	**0.001**
FVIII (%)	123.7± 51.0	169.6± 51.4	126.3 ± 17.2	**0.018**	0.848	**0.004**
FIX (%)	61.3± 25.0	48.7± 18.5	39.4 ± 10.3	0.217	**0.003**	**0.012**
FX (%)	53.3 ± 12.8	54.4 ± 12.6	64.3 ± 16.3	0.906	0.145	0.358
VWF Ag (%)	147.8 ± 26.0	233.2 ± 103.9	195.8 ± 51.8	**<0.001**	**0.003**	**0.024**
Rcof (%)	137.1 ± 23.6	169.9 ± 74.9	167.3 ± 30.0	**0.002**	**0.038**	**0.044**
Antithrombin (%)	63.1 ± 13.3	47.7 ± 10.2	68.5 ± 21.5	0.073	0.374	**0.011**
Protein C (%)	40.1 ± 8.3	28.4 ± 8.2	36.2 ± 10.1	**0.023**	0.222	0.162
Protein S (%)	44.5 ± 11.2	46.1 ± 23.4	56.4 ± 19.7	0.553	**0.034**	**0.010**
D-Dimers (μg/mL)	6.4 ± 7.5	3.2 ± 2.4	1.5 ± 1.0	0.171	**0.016**	**0.007**
PLT (×10^9^/L)		217.5 ± 97.8	419.4 ± 185.1			**0.002**
ADAMTS-13 (ng/mL)	577.2 ± 113.6	488.5 ± 75.4	618.8 ± 130.0	**0.015**	**0.002**	**0.004**

Data are expressed as mean values and standard deviation. *p* < 0.05 was considered statistically significant. Values presented in bold are statistically significant. P^1^: infection acute phase versus controls. P^2^: infection recovery versus controls. P^3^: infection acute phase versus recovery. Abbreviations: PT, prothrombin time; APTT, activated partial thromboplastin time; VWF Ag, von Willebrand factor antigen; Rcof, ristocetin cofactor activity; PLT, platelet count.

## Data Availability

The data presented in this study are available on request from the corresponding author. The data are not publicly available due to privacy.
